# Developing a Public Health Maternal and Child Health Training Program: Lessons Learned from Five Schools of Public Health

**DOI:** 10.1007/s10995-021-03327-9

**Published:** 2022-01-03

**Authors:** Christine T. Bozlak, Qiana L. Brown, Renee Davis, Rachel de Long, Melissa M. Howard, Teri E. Lassiter, Maria J. Perez-Patron, Brandie DePaoli Taylor, Renee Turchi, Michelle Menser Tissue

**Affiliations:** 1 Health Policy, Management, and Behavior, University at Albany School of Public Health, One University Place, Rm 173, Rensselaer, NY 12144 USA; 2 School of Social Work, Rutgers, The State University of New Jersey, 390 George Street, Suite 508, New Brunswick, NJ 08901 USA; 3 Department of Community Health & Prevention, Dornsife School of Public Health, Drexel University, 3215 Market St, 4th Floor, Philadelphia, PA 19104 USA; 4 Department of Health Promotion and Disease Prevention, Robert Stempel College of Public Health & Social Work, Florida International University, 11200 SW 8th Street, Miami, FL 33199 USA; 5 Diversity, Equity, & Inclusion, Urban-Global Public Health, Rutgers School of Public Health, 1 Riverfront Plaza, Suite 1020, 10th Floor, Newark, NJ 07076 USA; 6 Department of Epidemiology and Biostatistics, Texas A&M School of Public Health, 212 Adriance Lab Rd, College Station, TX 77843-1288 USA; 7 Obstetrics and Gynecology, Preventative Medicine & Community Health, Division of Basic & Translational Research, The University of Texas Medical Branch, MRB 11.158A, 301 University Blvd., Galveston, TX 77555 USA; 8 Pediatrics & Pediatrician-in-Chief, St. Christopher’s Hospital for Children, Center for Children and Youth With Special Healthcare Needs, Maternal and Child Health Program, Drexel University College of Medicine and Drexel Dornsife School of Public Health, 3215 Market Street, Philadelphia, PA 19104 USA; 9 Maternal and Child Health Bureau, 5600 Fishers Ln., Rockville, MD 20857 USA

**Keywords:** MCH workforce development, Graduate education, Schools of public health, MCH training program

## Abstract

Maternal and child health (MCH), as a core sub-field of public health, continues to be an essential area in which additional workforce development and investment are needed. Recent public health workforce assessments in the United States reveal there will be a significant number of vacancies in MCH public health positions in the near future, creating the need for a well-trained and skilled public health MCH workforce. In order to address this potential critical workforce gap, the U.S. Department of Health and Human Services, Health Resources and Services Administration’s Maternal and Child Health Bureau initiated the Maternal and Child Health Public Health Catalyst Program in 2015 to support the creation of MCH training programs in accredited schools of public health that previously did not have a MCH concentration. This article details the accomplishments and lessons learned from the first five MCH Catalyst Program grantees: Drexel University; Florida International University; Rutgers, The State University of New Jersey; Texas A&M University; and the University at Albany.

## Significance

This manuscript is the first to detail the accomplishments and lessons learned from the first five years of the HRSA Maternal and Child Health Bureau’s MCH Public Health Catalyst Program.

## Introduction

Maternal and child health (MCH), a sub-field of public health, continues to be an essential area in which workforce development and investment are needed. According to an Association of Maternal and Child Health Programs (AMCHP) analysis of the 2017 Public Health Workforce Interests and Needs Survey (PH WINS; de Beaumont Foundation, 2017), 24% of the MCH workforce surveyed plan to retire within the next five years, and the workforce, at all levels, indicated a need for additional MCH knowledge and skill development (AMCHP, n.d.). Consequently, an urgent need to fill these vacancies with a well-trained public health MCH workforce is anticipated. However, only 14% of the current state and local governmental MCH workforce has received formal public health training (AMCHP, n.d.). To appropriately address population-level MCH needs, a structure is needed to ensure the MCH workforce is adequately trained in public health and the MCH Leadership Competencies (U.S., 2018). MCH public health training programs provide one solution. This article will discuss initial outcomes and lessons learned from the first five Health Resources and Services Administration (HRSA)-funded MCH Public Health Catalyst Training Programs (referred to as “Catalyst Programs” and “Programs”) in the United States, which included the following institutions: Drexel University; Florida International University; Rutgers, The State University of New Jersey; Texas A&M University; and the University at Albany.

The Health Resources and Services Administration’s Maternal and Child Health Bureau (MCHB) has supported MCH Training Programs since the 1940s (Hutchins, 1994) and currently administers a continuum of workforce development programs, including academic training programs, to ensure the future and practicing MCH workforce is trained in the unique needs of MCH populations (US DHHS HRSA MCHB, n.d.a.). MCHB’s public health workforce development efforts are supported through funding from the Special Projects of Regional and National Significance, administered as part of Title V of the Social Security Act^1^ (US DHHS HRSA, n.d.). These efforts emphasize preparing future MCH professionals who are equipped to work in Title V agencies and to support academic-practice partnerships between MCH academic programs and state MCH agencies.

These programs have played a critical role in developing the MCH workforce infrastructure across the country, and MCH training programs’ impact, excluding the Catalyst Programs, has previously been documented (Kavanagh et al., 2015). While MCHB has supported MCH training in Schools of Public Health (SPH) over time, including the Centers of Excellence in MCH Education, Science and Practice Program (CoEs), recent workforce assessments highlight the need to expand MCH public health curriculum in Schools of Public Health and to train a diverse MCH public health workforce representative of the U.S. population (Association of Maternal and Child Health Programs. (n.d.a.); de Beaumont Foundation., 2017). In response to these identified workforce development needs, MCHB established the MCH Public Health Catalyst Program in 2015.

The five initial Catalyst Programs were awarded based on a competitive, objective review of applications in response to an MCHB-issued Notice of Funding Opportunity. With this program, the number of SPH with MCH public health content has increased, and there is expanded geographic diversity in MCH degree offerings. The Catalyst Programs also bring a strong emphasis on recruiting MCH students from racial and ethnic groups that are underrepresented in the MCH workforce. MCHB awarded each Catalyst Program up to $70,000 per year for five years. Each program was encouraged to pursue additional funding sources to build sustainability.

The MCH Public Health Catalyst Program goals are to: (1) develop foundational MCH content and competencies within SPH where no MCH concentration currently exists; (2) provide MCH content exposure to an increased number of public health students, introducing students to careers in the MCH field; and (3) recruit graduate students who are from backgrounds (including racial and ethnic minorities) underrepresented in the MCH field. The purpose of this article is to describe the facilitators and challenges of implementing an academic MCH program in accredited U.S. Schools of Public Health where a program did not already exist. This article will report on early successes, initial programmatic impacts, and lessons learned to inform other academic institutions that also aspire to integrate MCH into their curricula.

## Methods

Data reported to MCHB from the five initial Catalyst Programs funded from 2015 to 2020, and additional data provided from each program’s evaluation system, were analyzed. MCHB collects performance measure data from grantees annually through the Discretionary Grants Information System (DGIS) to assess progress in attaining program goals. This reporting mechanism has been the impetus for Catalyst Programs to create internal evaluation processes to demonstrate their impact and areas in need of enhancement.

Multiple measures of interest were analyzed based on Catalyst Program performance measure submissions to HRSA from Fiscal Years 2015–2019 and outcome data collected and reported by trainees in the post-training period. Outcomes of interest at two-years post-training include the percentage of Catalyst Program graduates demonstrating MCH field leadership after graduation, and the percentage of graduates engaged in work related to MCH populations. Field leadership is defined as providing MCH leadership within the clinical, advocacy, academic, research, public health, public health policy, or governmental realms in a variety of activities, but not necessarily managerial roles. An example of a public health practice leadership activity is procuring a grant or other funding in an MCH area.

Additional data collected through DGIS and aggregated across programs include the number of trainees at each institution and the diversity of trainees. Catalyst Program directors also supplied aggregated programmatic quantitative and qualitative data related to their trainees, infrastructure, administration, as well as reflections as program directors on challenges and barriers. This article is not based upon clinical study or patient data, and it was determined that Institutional Review Board approval was not needed for the collection of these programmatic data or for this article.

### Results/Initial Program Outcomes

The results and initial program outcomes from the first five years of funding for the initial Catalyst Programs are described below. Due to evaluation and data collection methods, trainee diversity and alumni data are only reported for 2015–2019.

### Program Structure and Curricula/Academic Programs

One specific goal of the Catalyst Program initiative was to ensure that foundational MCH content was delivered for at least nine months of every grant year (U.S., 2014). Within the first year (2015–2016), all Catalyst Programs developed foundational MCH courses covering the historical, legislative, and public health systems knowledge base regarding Title V and related programs in the United States. In one program, the foundational MCH course partnered with the state’s Title V program on a project related to current state and national MCH priorities.

MCH courses covered the empirical and theoretical basis for a life course perspective in addressing the unique developmental needs of MCH populations. A health equity and epidemiologic framework was used to understand these needs in the context of physical, social, and environmental influences and interactions. All programs addressed cultural and linguistic competency by stressing the importance of developing these skills for public health practice. The programs are grounded in the MCH Leadership Competencies (U.S., 2018), and informed by regional and national MCH needs assessments. Created in 2007, these competencies are a set of knowledge, skills, and personal characteristics that are designed to guide the professional development of all U.S. MCH professionals (U.S., 2018). Catalyst Programs used the competencies to develop curricula and professional development opportunities. Some programs continue to use them to assess individual trainee development. Although it was not a requirement of the training grant, all the programs developed MCH academic graduate certificates, concentrations, and/or a minor within the five-year grant period with a range of participation. At the end of the grant period, two programs had just begun to recruit students for their new academic programs and one program had enrolled 40 students.

The structure of the Catalyst Programs varied by institution. All programs included faculty who served as program director/co-directors. Affiliated faculty with MCH teaching and research interests were identified to expand the institution’s MCH network and strengthen trainee mentorship, research, and curriculum development. In total, the number of affiliated faculty ranged from 3 to 19 across each of the programs. Programs used a variety of methods to communicate with and use faculty expertise within their own institutions to support the development of their trainees, build a MCH learning community, and achieve their MCH program goals, including meetings, faculty work groups, and email listserv communications.

### Trainee Engagement

Given the uniqueness of each institution, trainee engagement looked different across programs and was tailored to meet trainee needs. From 2015 to 2020, Catalyst Programs supported MCH trainees in a variety of ways, including funding for: 1) travel to attend MCH-related conferences; 2) teaching assistantships for MCH courses; 3) “Scholar Consultant” opportunities to consult on the MCH program and its courses by providing regular feedback while also participating in community-based participatory research projects; and 4) Stipends to provide tuition-only support for the students’ MCH coursework or completion of an MCH-focused internship. Each program developed a MCH-focused student interest group or advisory committee. The groups’ structure varied with some programs meeting two to three times per year while others met bi-monthly.

Across the five institutions and five-year timeframe, 138 trainees were awarded individual funding to support their MCH professional development. At least 615 funded and unfunded trainees across all five programs had at least one contact hour (the minimum contact needed to be considered a MCH trainee) with the Catalyst Programs through programmatic activities from 2015 to 2020. These trainees engaged in activities, such as MCH-related seminars, journal clubs, interest groups, open houses, symposia, MCH courses, MCH academic programs, internships, listservs, an MCH app, mentoring, and funding opportunities for MCH professional development. Trainees and faculty also jointly produced publications, conference presentations, and policy briefs.

Catalyst trainees from all programs extensively engaged with their communities and public health systems serving MCH populations. Programs engaged trainees in over 120 collaborative activities with state Title V agencies and other community MCH partners, such as continuing education open to professionals and trainees, advocacy, peer education, and research. Programs hosted outreach and awareness events on infant mortality, sexual health, alcohol and opioid use in pregnancy, and other MCH topics. Trainees volunteered at MCH organizations and hosted clothing and toiletry drives for MCH causes, co-hosted training events with community partners, conducted MCH research with faculty on issues including maternal and infant health, asthma, stillbirth prevention, and food insecurity. Trainees mentored other MCH-focused students, including peers at the same educational level or below, through informal discussions, guest lectures in undergraduate MCH courses, and the creation of training opportunities for their peers.

At the state level, three Catalyst programs engaged with their state Title V programs. As new training programs, Catalyst Programs were expected by HRSA to make initial outreach to their state’s Title V program to begin to build a relationship, but not to provide technical assistance. Collaborations included development of a maternal mortality prevention webcast, student recruitment for Title V placements, partnership on MCH course projects, Title V staff presentations, and technical assistance for the state’s Title V needs assessment. At the national level, sixteen Catalyst trainees, representing all five Catalyst programs, participated in the Title V MCH Internship Program from 2017 to 2020, providing increased exposure to Title V careers for Catalyst trainees (Handler et al., 2018). All Catalyst Programs included Title V educational content in their curricula.

### Trainee Recruitment and Diversity

One of the Catalyst Program’s objectives is to recruit graduate students from backgrounds underrepresented in the MCH field. Ensuring a well-prepared, diverse and culturally competent workforce is a key strategy to reduce health disparities and to improve health outcomes (Agency for Healthcare Research & Quality, 2018). Programs used various trainee recruitment methods for engagement with their programs, especially among diverse trainees. Some of these methods included: leveraging existing recruitment activities within their institutions to advertise trainee opportunities, developing a scholarship program which provided funds for MCH-related research and practicum opportunities, holding recruitment open houses, and recruitment through student listservs and newsletters.

Catalyst Programs were situated within existing diverse higher education institutions that also have scholarships to recruit diverse students. Diversity recruitment efforts were closely linked with those same efforts of the program’s SPH, which often included attending public health events, such as the American Public Health Association’s annual meeting, for recruitment purposes. Existing undergraduate public health programs and graduate online public health programs also helped to increase the programs’ diversity. Although this is an area in need of greater attention at most institutions, engaging diverse faculty within each program from various disciplines and identities through guest lectures in MCH courses, mentoring relationships for MCH trainees, and MCH research opportunities with trainees, also helped to recruit diverse trainees.

As demonstrated in Table 1., for the reporting periods between 2015 and 2019, 56.5% of Catalyst trainees were from an underrepresented racial or ethnic group, which is twice as many as the 27% of the U.S. population who self-identified as non-white during this timeframe (U.S. Census Bureau, 2019). In comparison, a 2016 Title V Workforce Assessment indicated that 76% of the Title V workforce identified as white (Association of Maternal & Child Health Programs, 2016). Hispanic/Latinos are the largest ethnic minority in the U.S., and comprised 18% of the population (U.S. Census Bureau, 2019) and 17% of Catalyst trainees in FY19. Goodman et al. (2020) reported that in 2016, 11.9% of public health masters students and 9.9% of doctoral students identified as Hispanic, respectively. These data are an indication that the Catalyst Programs show promise in helping to build a more diverse public health and MCH workforce that is representative of the U.S. population.

**Table 1:** Aggregate Catalyst program race and ethnicity trainee data (2015–2019)

Year	Total trainees that were engaged in MCH activities including coursework, practicum experiences, and student interest group activities during a 12-month reporting period (beyond 39 contact hours)	Percent of trainees identifying as being from an underrepresented racial group	Percent of trainees identifying as Hispanic or Latino
2015	72	39 (54.2%)	12 (16.7%)
2016	94	51 (54.3%)	15 (16.0%)
2017	93	58 (62.3%)	12 (12.9%)
2018	124	66 (53.2%)	11 (8.9%)
2019	102	60 (58.9%)	17 (16.7%)
2015–2019	485	275 (56.5%)	67 (13.8%)

### Initial Trainee Post-Graduation MCH Career Outcomes

In FY19, 89% of former trainees from the five Catalyst Programs demonstrated leadership in MCH two years after program completion in any of the following areas: academic, clinical, public health practice, and public policy and advocacy. Former trainees most frequently reported academic or public health practice leadership. Sixty-five percent of former trainees indicated they were working with MCH populations.

Through mentorship, direct linkages with external partners, networking events, job postings in e-newsletters, and providing formal recommendations, former trainees secured full-time positions in local community-based organizations (e.g. child abuse prevention organization, child care coordinating council), state Title V programs, and national MCH organizations (e.g. American College of Obstetricians and Gynecologists, March of Dimes). Former trainees are also working in federal agencies, including HRSA, the National Institutes of Health, the Centers for Disease Control and Prevention, and the Department of Veterans Affairs. Please see Table 2. for the post-graduation trainee placements of each program’s trainees. Several former trainees continued with their formal education pursuing clinical degrees in nursing and medicine, doctoral degrees, and professional MCH-related certifications (e.g. doula and lactation certifications). Trainees also continue to serve their professional communities, such as the APHA MCH Section and the Association of Teachers of MCH (ATMCH).

**Table 2:** Post-graduation MCH-related placement of trainees by Catalyst program

Drexel University	Academic organization
	Clinical organization/health care setting
	Other federal agency
	Local health department
	MCH advocacy organization
	Community-based organization
Florida International University	Academic organization
	Clinical organization/health care setting
	Other state agency
	Local health department
	Community-based organization
	Research institution
	Other: Nursing; PhD in Public Health programs
Rutgers University	State Title V program
	Local health department
	MCH advocacy organization
	Community-based organization
Texas A&M University	Academic organization
	Clinical organization/health care setting
	HRSA
	Other federal agency
	State Title V program
	Local health department
	MCH advocacy organization
	Community-based organization
	Research institution
	Other: International organization
University at Albany	Academic organization
	Clinical organization/health care setting
	HRSA
	State Title V program
	Other state agency
	Local or city health department
	MCH advocacy organization
	Community-based organization
	Research institution
	Other: Consulting firm; K-12 schools; Professional association

## Discussion and Conclusion

In 2015, the establishment of the HRSA MCH Public Health Catalyst Program resulted in the creation of five MCH Programs at Schools of Public Health throughout the United States where there was previously limited MCH curricula. These programs differ in their structure, operation, curricula, and professional development opportunities. However, the programs share commonalities in purpose and passion to expand the United States’ MCH workforce and develop MCH leaders. These initial Catalyst Programs benefitted from having this training grant to support their creation in a coordinated manner, while also benefitting from the mentorship of the HRSA MCHB and the CoEs. HRSA MCHB and the COEs engaged in conference calls and annual grantee meetings with the Catalyst Programs to share best practices pertaining to curriculum development, trainee engagement, diversity and inclusion, and Title V engagement.

This article is limited by the short duration of data included (2015–2019). Despite still being in a formative state and needing to collect long-term outcome data, the initial impact of these MCH programs has been substantial. The programs have also experienced individual challenges in their development including changes in leadership and also with their state Title V workforce that make it difficult to establish and maintain partnerships. Programs have encountered challenges with differing levels of trainee leadership and engagement across academic years for their MCH student organizations.

The following are lessons learned and recommendations for other schools and programs of public health seeking to establish MCH programs or curricula. The field of MCH is multidisciplinary and interdisciplinary. MCH academic programs must engage other disciplines to support the diversity in MCH student interests. These efforts can also help with long-term program sustainability. For example, other schools outside of, but complementary to public health, may contribute resources and expertise if their students are engaged in MCH efforts. Similarly, programs should have a continued focus on establishing and maintaining strong partnerships with local MCH organizations and state Title V programs as a key element of their MCH programs. External partners can also help secure funding to diversify funding streams for the program.

Sustainability must be built into the program at its inception, which includes working with institutional leadership to formalize the program into the organizational structure expeditiously. The Catalyst Programs used the initial HRSA investment to begin their MCH programs and to leverage other funds by local foundations and state agencies. One Catalyst Program established a memorandum of understanding with the state Title V program to support funded trainee internships in the Title V program.

New MCH programs are encouraged to reach out to existing MCH programs at other institutions for mentorship and best practices, as well as attend MCH-focused conferences (e.g. AMCHP) for information and networking. For example, ATMCH provides free membership, curriculum resources on their website, and provided mentorship and Innovation in MCH Teaching awards to two of the Catalyst Programs to assist these programs with the development of their curricula. In addition to these lessons learned, Table 3. includes recommendations for institutions wanting to establish MCH Programs.

**Table 3:** *Recommendations for other schools and programs of public health for MCH program initiation

During program development	During program implementation
Talk with other SPH with a MCH Program	Focus on publicity of program
Assess institution for MCH-focused faculty, staff, courses, and related programs	Use resources, such as ATMCH, AMCHP, and MCH Navigator
Gain support of administrators	Leverage the resources/expertise of institution
Establish collaborations with local organizations and the state Title V program	Hire a program coordinator
Consider multiple sources of funding	Provide students with an opportunity for leadership, but be selective – only the most motivated students are able to lead others
Build the student pipeline through undergraduate and other academic programs	Implement a clear evaluation plan

^*^Initially presented as a poster presentation at the 2019 Association of Maternal and Child Health Programs conference

In summary, the HRSA MCH Public Health Catalyst Program grant was critical in garnering institutional and administrative support and initiating MCH programs at the five institutions featured in this article. In 2021, de Beaumont et. al. (2021) reported there is a need for 80,000 more full-time positions within state and local health agencies to meet community needs. The 2017 PH WINS survey indicated that more than a third of respondents from state health agencies were considering leaving their agency within the next year (Halverson, 2019). During this time when U.S. MCH populations are at increased risk for negative health outcomes and with heightened visibility of existing health disparities in these populations due to COVID-19 (Dongarwar et al., 2020; Hill & Burroughs, 2020), it is imperative that Schools of Public Health continue to recognize the importance of MCH as a core field within public health and work to secure the future, growth, and diversity of the MCH workforce. HRSA MCHB’s investment in the MCH Public Health Catalyst Program represents a promising effort to help address these persistent MCH workforce needs.

## Data Availability

Not applicable.

## Footnotes

1 The Title V MCH Block Grant Program is a formula grant under which funds are awarded to 59 states and jurisdictions to support the health and well-being of all mothers, infants, and children, which includes infants and children with special health care needs (CSHCN), and their families. (US DHHS HRSA MCHB, n.d.b.).
